# Professional content analysis and quality assessment of cardiopulmonary resuscitation educational videos on social media platforms: a comparative study of YouTube, BiliBili, and TikTok

**DOI:** 10.3389/fpubh.2025.1657233

**Published:** 2025-09-15

**Authors:** Chuanliang Pan, Luping Cheng, Bo Zhang, Xia Hu, Wenxin Wang, Guanglin Jiang

**Affiliations:** ^1^Department of Intensive Care Unit, The Third People’s Hospital of Chengdu, Affiliated Hospital of Southwest Jiaotong University, Chengdu, China; ^2^School of Clinical Medical, North Sichuan Medical College, Nanchong, China

**Keywords:** cardiopulmonary resuscitation, social media, public education, public health, information quality, PEMAT, VIQI, GQS

## Abstract

**Background:**

Cardiopulmonary resuscitation (CPR) is an emergency medical procedure designed to restore circulation and respiratory function in patients who have suffered cardiac arrest. This study aimed to comprehensively analyze the upload sources, content characteristics, and video quality of CPR-related videos on YouTube, Bilibili, and TikTok, with a view to providing a reference for improving public first aid awareness and skills.

**Methods:**

In December 2024, we searched each platform using “Cardiopulmonary resuscitation” and “CPR” (including “心肺复苏” for Bilibili and TikTok), retrieving the top 100 videos per platform. After screening, 239 videos (YouTube: 80; Bilibili: 72; TikTok: 87) met inclusion criteria. Meanwhile, we quantitatively assessed the video quality using the Patient Education Material Assessment Tool (PEMAT), Video Information and Quality Index (VIQI), and Global Quality Score (GQS) assessment tools. We assessed the correlation between video quality scores and viewer interaction data (likes, comments, favorites, and retweets).

**Results:**

A total of 239 videos were included for analysis (YouTube: 80; Bilibili: 72; TikTok: 87). Short-form CPR videos have increased yearly. Uploaders differed by platform: YouTube—mainly professional institutions; Bilibili—Non-professional individuals; TikTok—Non-professional Institutions. TikTok had the highest uploader certification rate (72.97%), and videos by professional individuals gained the most interactions. Content varied: YouTube focused on CPR knowledge (85.00%), TikTok on News and Reports (48.28%), and Bilibili was mixed. The automated external defibrillator (AED)-related videos on TikTok received the most likes. YouTube videos had the highest quality scores, especially those from professionals. However, correlation between quality scores and interaction data showed no strong positive correlation.

**Conclusion:**

Social media plays a growing role in CPR education, yet overall video quality—especially in accuracy and completeness—needs improvement. Involving more professionals in content creation and enhancing platform recommendation algorithms could help disseminate reliable first aid information more effectively.

## Introduction

Cardiopulmonary resuscitation (CPR) is one of the most important first aid skills in acute and critical care medicine (1) and is widely used in the emergency resuscitation of patients with cardiac arrest, which is decisive for saving lives. Cardiac arrest is a sudden, life-threatening emergency that manifests itself as a sudden cessation of heartbeat and respiration. The main goal of CPR is to restore blood circulation to the brain and other vital organs by means of chest compressions and artificial ventilation until autonomic circulation can be restored or further specialized medical support can be obtained (2). Studies have shown that the use of CPR is considered a potentially life-saving measure, and that effective early intervention is essential to improve the survival of cardiac arrest patients (3–5). Prompt administration of high-quality chest compressions and respiratory support within the first few minutes after the onset of cardiac arrest significantly improves the patient’s chances of survival (6). Although cardiac arrest can occur in all age groups, the older adults those with underlying cardiovascular disease, and those with cardiac arrest due to accidents such as drowning and electrocution are among the high-risk groups. In addition, Ashish et al. suggested (7) that whether or not to initiate bystander CPR (B-CPR) is also influenced by a variety of factors, including demographic characteristics, subjective willingness, knowledge base, skill level, and scene environment. Although many bystanders possess the willingness to rush, they are often unable to perform effectively due to a lack of basic knowledge or operational skills (8).

Although CPR is a highly technical first aid measure, its basic operation is relatively simple and can be mastered by the general public through standardized training. However, due to the practical application, there are often problems such as insufficient compression, substandard frequency, or poor ventilation, which affect the resuscitation effect (9). In addition, CPRCPR operation may also bring certain risks, such as chest injury or rib fracture, especially in older adults patients. Therefore, standardized training and hands-on practice are essential to improve the quality of CPR, optimize the resuscitation outcome, and reduce the risk of related complications.

In recent years, with the rapid development of digital technology, online video platforms are becoming an important way for the public to obtain health information (10). Compared with traditional health education, the Internet provides patients with greater initiative, enabling them to take the initiative to search and learn health knowledge through online search, thus improving their health management ability (11). However, health information disseminated on short-form video platforms is uneven, and some of the content contains medical errors that may lead to public and trigger inappropriate decision-making (12, 13). Therefore, it is of great practical significance to improve the scientificity and credibility of online health information.

Currently, all major video platforms feature a large number of short videos related to CPR, with YouTube, Bilibili, and TikTok being the most common (14–16). Previous studies have analyzed the quality of videos on topics such as Cardiac Rehabilitation (17), dermatology (18), Coronavirus Disease 2019 (COVID-19) (19), and diabetes (20), but there is still a lack of systematic evaluation of the quality of CPR-related videos on these platforms. Therefore, this study aims to investigate and analyze the quality of CPR-related video content on YouTube, Bilibili, and TikTok, with the goal of providing scientific guidance for the public to learn CPR online, as well as offering constructive suggestions to content creators and platform regulators.

## Materials and methods

### Ethical considerations

All information comes from publicly available YouTube, TikTok, and Bilibili videos, and does not involve any personal privacy issues. The study did not use clinical data, human samples, or laboratory animals. Therefore, ethical review is not required.

### Search strategy and data collection

The video search was conducted on January 6, 2025. To minimize interference from personalized recommendation algorithms, we prepared new devices before retrieval and did not log into any related accounts. On the YouTube platform, the keywords “Cardiopulmonary Resuscitation” and “CPR” (this is both a scientific name and a common colloquial term) were used for retrieval; on the Bilibili and TikTok platforms, the Chinese keyword “心肺复苏” and the English “CPR” were used, respectively. No filtering conditions were set during retrieval, and videos were arranged in the default sorting order. Considering the stability of video exposure, attention, likes, and comments, videos published within the past week were excluded, and the retrieval cutoff date was set as December 30, 2024. Ultimately, the top 100 ranked videos on each platform were selected for subsequent analysis (21, 22), with videos of an advertising nature excluded. Subsequently, videos were screened according to the following exclusion criteria: (1) duplicate content uploaded by different uploaders; (2) videos missing uploaders information or video titles; (3) videos unrelated to cardiopulmonary resuscitation; (4) videos related to animal cardiopulmonary resuscitation; (5) videos without subtitles and without audio.

### Video and uploader characteristics

For the videos included in the analysis, the data we collected included the upload platform, video title, uploader’s identity (account name, self-description, certification status, number of followers), upload time, video lengths, number of views, likes, comments, favorites, and shares. However, it should be noted that TikTok does not provide the number of views, and YouTube lacks data on favorites and shares. All collected data were recorded in Excel (Microsoft Corporation). We categorized video uploaders based on their identity into Professional Individuals, Non-professional Individuals, Professional Institutions, and Non-professional Institutions. Among Professional Individuals, we further divided them into Doctors Specializing in Emergency Medicine, Doctors in Other Fields of Modern Medicine, First Aiders and Care Personnel, and Other Healthcare Professionals. The content categories of the videos are as follows: (1) CPR Knowledge, (2) Case Analysis and Practice, (3) Application of Automated External Defibrillators (AEDs), (4) CPR Skills Challenge Footage, (5) News and Reports. For specific classification criteria (Multimedia Appendix 1 in Supplementary material).

### Video evaluation

Three evaluators independently rated each video using the following three tools, and inter-rater reliability among the three independent raters was assessed using the intraclass correlation coefficient (ICC). In case of discrepancies in the ratings, the team members discussed together to determine the final score. Video quality assessment was conducted from three dimensions: information quality, content quality, and overall quality. Information quality mainly examined the fluency and accuracy of the information, assessed using the Video Information and Quality Index (VIQI). Content quality focused on the reliability and completeness of the video content, evaluated using the Patient Education Materials Assessment Tool (PEMAT). Overall quality integrated the above aspects to assess the video’s usefulness to viewers, using the Global Quality Score (GQS). All of the above tools have been validated in previous studies (16, 23).

The specific scoring methods and characteristics of each assessment tool are as follows (Multimedia Appendix 2 Supplementary material).

VIQI (24) can comprehensively analyze the overall quality of a video from multiple dimensions, including four scoring criteria: information flow (VIQI 1), information clarity (VIQI 2), video quality (VIQI 3), and consistency (match between the title and content of the video) (VIQI 4). Each item is rated on a 5-point Likert scale, ranging from 1 to 5 (1 = poor quality and poor fluency, 2 = overall poor quality and poor fluency, 3 = moderate quality and suboptimal fluency, 4 = good quality and overall fluency, 5 = excellent quality and fluency). Higher scores of VIQI total score (VIQI-sum) indicate better quality.

The PEMAT was developed in 2014 and has good internal consistency, reliability and construct validity. The tool contains 25 items, of which 21 assess the understandability of the health information and 4 assess the actionability of the recommended measures. The scoring format is “Agree = 1, Disagree = 0, Not Applicable = Not Counted.” The total score (PEMAT-T), understandability score (PEMAT-U), and actionability score (PEMAT-A) were calculated as “sum of scores/total possible scores×100%.” A score of ≥70% indicates that the information is easy to understand or has actionable guidance (23).

GQS (14) is a 5-point scale, with scores ranging from 1 (poor) to 5 (excellent), which comprehensively evaluates the clarity, authority, and educational value of video information. It is suitable for rapid overall quality assessment. The higher the score, the more reliable and comprehensive the video content.

### Statistical analysis

We used IBM SPSS Statistics version 24.0 (IBM Corp., Armonk, NY, United States) for statistical analysis of the data. The Shapiro–Wilk test was used to assess the normality of continuous variables. For normally distributed continuous variables, results are presented as mean ± standard deviation (mean ± SD); for non-normally distributed continuous variables, results are presented as median (*M*), quartile distribution (IQR, [P25, P75]), and minimum-maximum (Min-Max) range. The Mann–Whitney U test was used for comparisons between groups. For comparisons among three or more independent samples with non-normal distribution, the Kruskal–Wallis test was used. Categorical variables are presented as frequency (n) and percentage (%), and comparisons among groups were performed using the Chi-square test or Fisher–Freeman–Halton test. For significant differences found in multiple group comparisons, the Bonferroni method was used for *post hoc* pairwise comparisons. We used Spearman correlation analysis to assess the correlation between audience interaction indicators (such as likes, comments, etc.) and video quality scores. The strength of the correlation was expressed using Spearman’s rank correlation coefficient (*r*), where *r* > 0 indicates a positive correlation and *r* < 0 indicates a negative correlation. |*r*| ≤ 0.2 indicates no correlation; 0.2 < |*r*| ≤ 0.4 indicates a weak relationship; 0.4 < |*r*| ≤ 0.6 indicates a moderate relationship; 0.6 < |*r*| ≤ 0.8 indicates a strong relationship; and |*r*| > 0.8 indicates a very strong relationship. A value of *p* < 0.05 was considered statistically significant.

## Results

### Video characteristics

**Figure 1:**
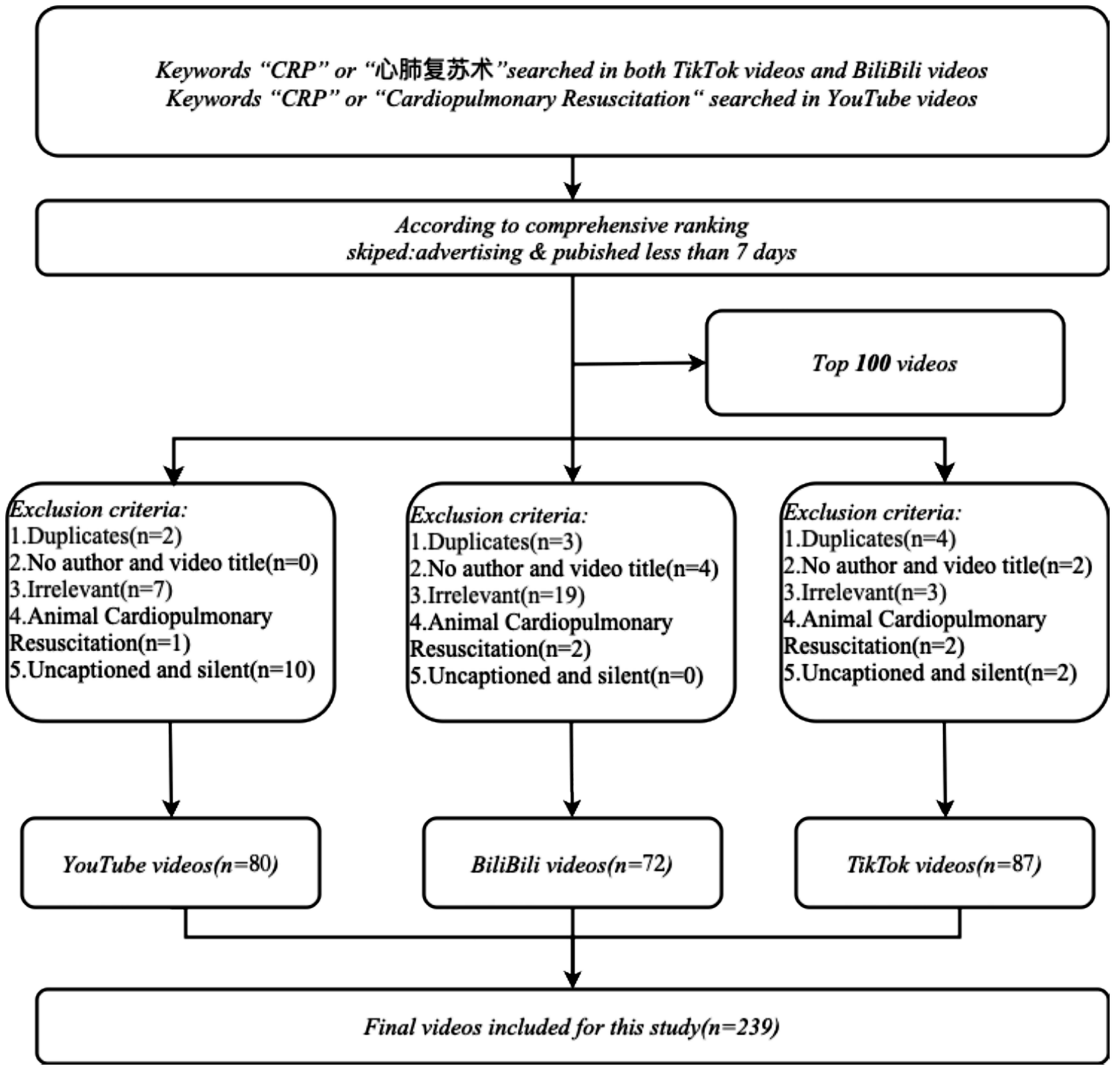
Flowchart of filtering CPR.

**Figure 2:**
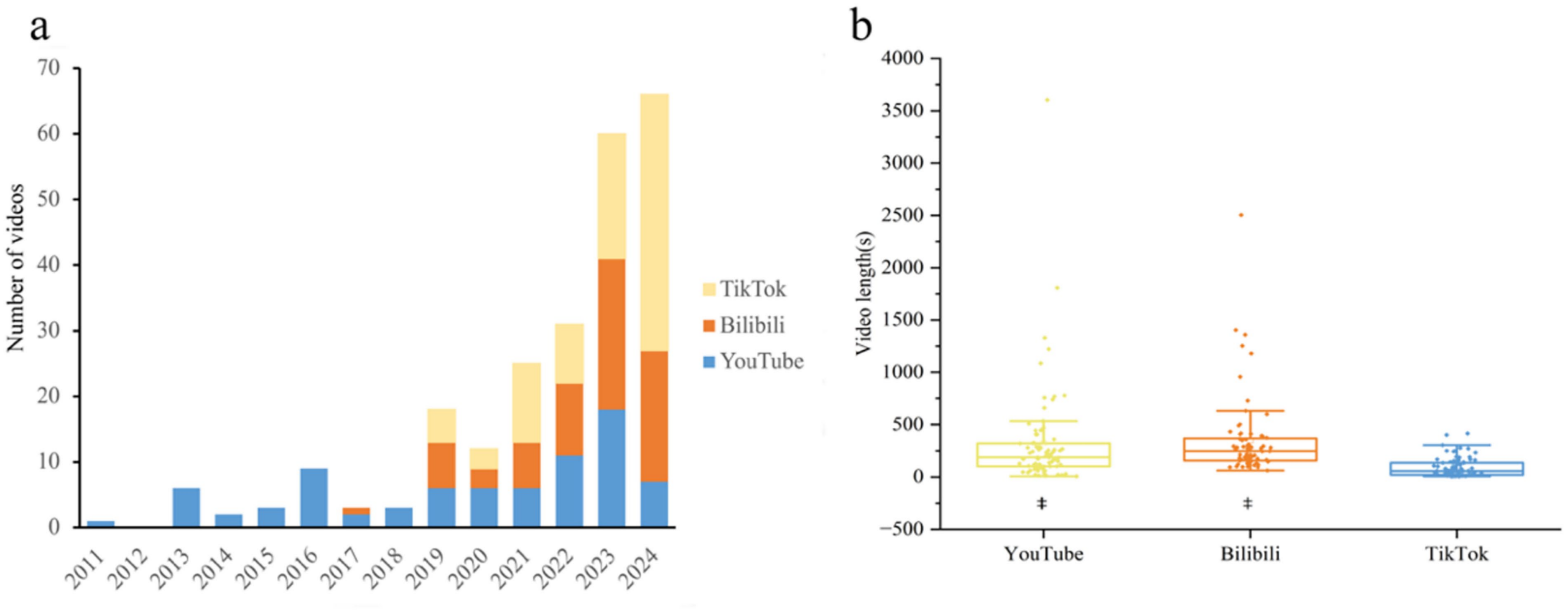
General information about CPR on YouTube/Bilibili/TikTok. **(a)** Annual distribution of eligible videos (*n* = 239). **(b)** Comparison of CPR video lengths on three platforms. ^‡^*p* < 0.05 vs. TikTok.

A total of 239 videos were included in this study, comprising 80 videos from YouTube, 72 from Bilibili, and 87 from TikTok. According to the preset inclusion and exclusion criteria, duplicate, irrelevant, and subtitle-free videos that did not meet the requirements were excluded (Figure 1). The videos included in the analysis were uploaded between 2011 and 2024 (Figure 2a). From 2011 to 2024, the year with the highest number of CPR-related video uploads on both YouTube and Bilibili was 2023, with 18 videos (22.50%) and 23 videos (31.94%) respectively; on TikTok, the year with the most videos was 2024, with 39 related videos uploaded (44.83%). In terms of video lengths, videos on YouTube and Bilibili were significantly longer than those on TikTok, with statistically significant differences among the three platforms (*p* < 0.001) (Figure 2b), which may be influenced by the content upload time limits of each platform. The video lengths for each platform were as follows: the 80 videos on YouTube had 186.0 (99.0, 3603.0) seconds; the 72 videos on Bilibili had 248.0 (156.5, 2504.0) seconds; and the 87 videos on TikTok were significantly shorter, at 57.0 (21.0, 416.0) seconds. The specific characteristics of the videos on each platform are shown in Table 1.

**Table 1:** Characteristics of videos about CPR on YouTube/Bilibili/TikTok.

YearRange	Platform	YouTube (Nl = 80)			Bilibili (N2 = 72)			TikTok (N3 = 87)		
	Characteristic	*M*	(Min, Max)	P25–P75	*M*	(Min, Max)	P25–P75	*M*	(Min, Max)	P25–P75
2011–2015	Views	340570.50	(2,448, 19,416,600)	46152.00–3718137.75	–	–	–	–	–	–
	Likes	1105.00	(4, 56,000)	185.00–28250.00	–	–	–	–	–	–
	Favorites	–	–	–	–	–	–	–	–	–
	Shares	–	–	–	–	–	–	–	–	–
	Comments	32.00	(0, 269)	1.00–155.50	–	–	–	–	–	–
	Video length(s)	109.00	(18, 320)	52.75–227.25	–	–	–	–	–	–
	Duration (day)	4111.00	(3,350, 4,890)	3609.25–4237.75	–	–	–	–	–	–
2016–2020	Views	84805.00	(119, 7,718,702)	2969.50–880451.00	35000.00	(4,013, 1,012,000)	7137.00–382,000.00	–	–	–
	Likes	575.50	(0, 65,000)	8.75–7969.25	287.00	(33, 18,000)	225.00–4082.00	17,500.00	(163, 3,157,000)	2488.50–25500.00
	Favorites	–	–	–	702.00	(79, 27,000)	510.00–5455.00	2641.00	(53, 16,000)	598.00–8713.25
	Shares	–	–	–	332.00	(32, 24,000)	220.00–3883.00	8885.50	(63, 17,000)	1564.00–15500.00
	Comments	41.00^a^	(0, 1,169)	0.00–166.50	19.00	(3, 861)	11.00–135.00	371.00	(5, 74,000)	84.25–1520.25
	Video length(s)	23850.00	(60, 1,221)	127.50–406.25	291.00	(147, 491)	175.00–387.00	59.00	(18, 416)	59.00–92.50
	Duration (day)	2433.50	(1,563, 3,252)	1862.00–3039.75	1860.00	(1,518, 2,749)	1748.00–2045.00	1858.00	(1,494, 2,172)	1528.50–1944.75
2021–2014	Views	88869.00	(70, 17,746,537)	4697.00-352432.00	31,000.00	(467, 1,968,000)	11000.00-77500.00	–	–	–
	Likes	777.50	(0, 1,350,000)	61.00–4348.25	237.00	(3, 75,000)	59.00–744.00	37000.00	(39, 2,713,000)	6243.00–134000.00
	Favorites	–	–	–	495.00	(8, 46,000)	130.00–1341.00	2610.00	(27, 465,000)	693.00–17000.00
	Shares	–	–	–	64.00	(0, 44,000)	6.50–564.50	4072.00	(7, 771,000)	924.00–26000.00
	Comments	22.00^b^	(0, 3,386)	1.00–136.00	12.00	(0, 4,654)	2.50–53.50	2120.00	(1, 197,000)	238.00–9289.00
	Video length(s)	186.00	(7, 3,603)	72.50–324.00	215.00	(61, 2,504)	147.50–368.00	53.00	(7, 402)	21.00–136.00
	Duration (day)	721.50	(96, 1,440)	495.00–850.50	565.00	(10, 1,410)	251.00–836.50	371.00	(1, 1,447)	173.00–770.00

a Excluded 2 YouTube videos with disabled comments.

b Excluded 3 YouTube videos with disabled comments.

CPR, Cardiopulmonary Resuscitation.

### Uploader and content characteristics

**Figure 3:**
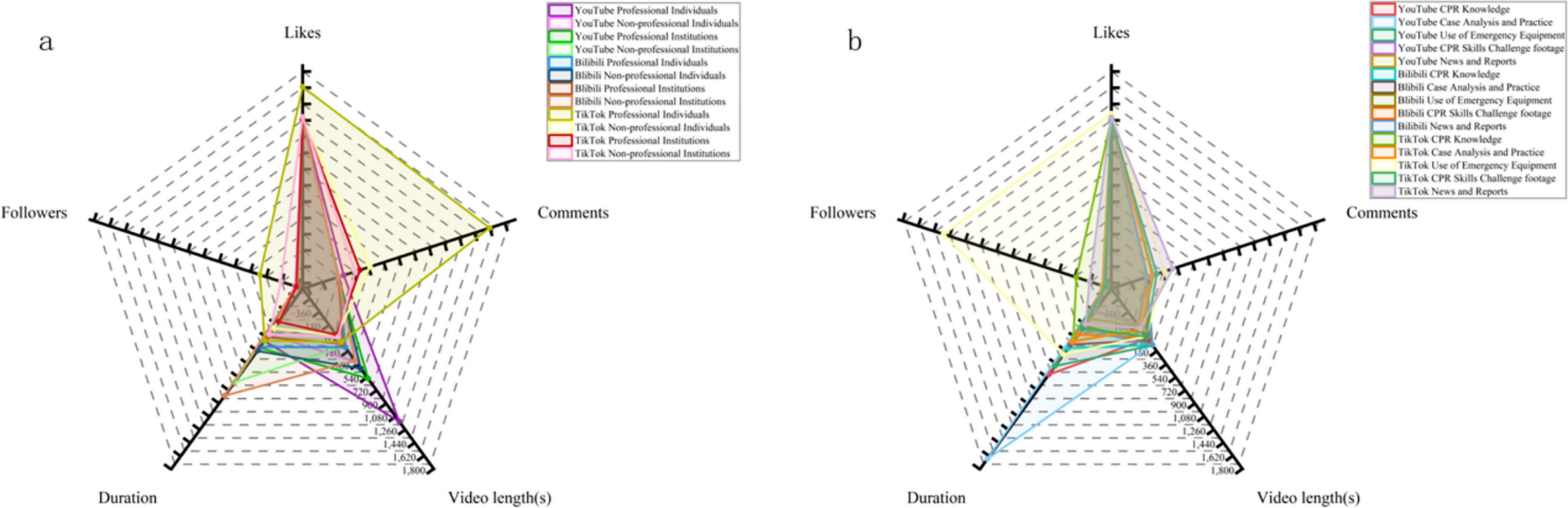
Comparative analysis of viewer interaction across three platforms by uploaders and video content. Excluded 5 YouTube videos with disabled comments. **(a)** Interaction of video uploaders. **(b)** Interaction of video content.

As shown in Table 2, there are 73, 64, and 74 video uploaders on YouTube, Bilibili, and TikTok, respectively. TikTok uploaders have the largest number of followers and the highest certification rate, with 54 certified uploaders (72.97%). There are significant differences in the type of video uploaders across platforms: YouTube uploaders are mainly Professional Institutions, Bilibili is dominated by Non-professional Individuals, while TikTok is primarily Non-professional Institutions. Despite the clear differences in uploader categories, the distribution of uploader among certified professionals is similar across platforms. Among them, Doctors Specializing in Emergency Medicine account for the highest proportion of certified uploaders on TikTok. After reviewing the 239 videos that met the inclusion criteria, we categorized their content into five types: (1) CPR Knowledge, (2) Case Analysis and Practice, (3) Application of AED, (4) CPR Skills Challenge Footage, and (5) News and Reports. There are significant differences in the content composition of videos across the three platforms (Table 2): YouTube videos are mainly focused on CPR Knowledge, accounting for 85.00%; Bilibili’s content is more dispersed, with CPR Knowledge (30.56%) and News and Reports (30.56%) as the main categories; on TikTok, News and Reports account for the highest proportion (48.28%). Notably, there were no videos related to CPR Skills Challenge Footage on YouTube. Among videos involving CPR content for children or infants, YouTube had the highest number, with a total of 13 videos (16.25%); Bilibili had 1 (1.39%); and there were no such videos among those included from TikTok. In terms of interaction, videos uploaded by Professional Individuals on TikTok received the most likes and comments. From a content perspective, videos related to AED on TikTok received the highest number of likes (*M* = 115,500), followed by TikTok News and Reports (*M* = 52,500) (Figure 3).

**Table 2:** Characteristics of uploaders and video content about CPR on YouTube/Bilibili/TikTok.

Platform	YouTube (N1 = 80)	Bilibili (N2 = 72)	TikTok (N3 = 87)	*P*
Number of uploaders	73	64	74	–
Followers, Median (P25, P75)	165500.0 (15900.0, 527500.0)^†‡^	1233.0 (171.5, 14000.0)^‡^	1,101,000.0 (35000.0, 7697000.0)	<0.001^a^
Certification, *n* (%)	39 (52.70%)^†‡^	10 (15.63%)^‡^	54 (72.97%)	<0.001^b^
Type of uploaders, *n* (%)				<0.001^b^
Professional individuals	9 (12.33%)	11 (17.19%)	13 (17.57%)	
Non-professional individuals	8 (10.96%)^†^	53 (82.81%)^‡^	20 (27.03%)	
Professional institutions	33 (45.21%)^†^	3 (4.69%)^‡^	12 (16.22%)	
Non-professional institutions	30 (41.10%)^†^	5 (7.81%)^‡^	42 (56.76%)	
Classification of certified professionals, *n* (%)				0.2634^c^
Doctors specializing in emergency medicine	1 (1.37%)	1 (1.65%)	5 (6.76%)	
Doctors in other fields of modern medicine	6 (8.22%)	6 (9.38%)	6 (8.11%)	
First aiders and care personnel	2 (2.74%)	1 (1.65%)	2 (2.70%)	
Other healthcare professionals	0	3 (4.69%)	0	
Type of content, *n* (%)				<0.001^b^
CPR knowledge	68 (85.00%)^†‡^	22 (30.56%)	26 (29.89%)	
Case analysis and practice	8 (10.00%)^†^	18 (25.00%)	16 (18.39%)	
Application of AED	11 (13.75%)^‡^	7 (9.72%)^‡^	2 (2.30%)	
CPR skills challenge footage	0^†^	6 (8.33%)	5 (5.75%)	
News and reports	4 (5.00%)^†^	22 (30.56%)^‡^	42 (48.28%)	
Pediatric/Infant CPR *n* (%)	13 (16.25%)^†‡^	1 (1.39%)	0	<0.001^c^

^†^*P* < 0.05 vs. Bilibili; ^‡^*P* < 0.05 vs. TikTok. In this study, Certified Professionals refer to Professional Individuals who have undergone identity certification, to distinguish them from Professional Institutions.

a Kruskal-Wallis test.

b Chi-squared test.

c Fisher–Freeman–Halton test.

CPR, Cardiopulmonary Resuscitation; AED, Automated External Defibrillator.

### Quality analysis

We evaluated the quality of videos included from the three video platforms according to established scoring criteria. Overall: the PEMAT-T score for all videos was 67.0 (34.0–100.0), the VIQI-sum score was 13.0 (11.0–15.0), and the GQS score was 4 0.0 (3.0–5.0). Statistical analysis showed that videos on the YouTube platform scored significantly higher than those on Bilibili and TikTok in both PEMAT-T and VIQI-sum, with statistically significant differences. Although there was no statistically significant difference in the GQS scores among the three platforms, the IQR showed that YouTube’s GQS scores were still higher than those of the other two platforms. From the overall trend, YouTube videos were of higher quality than those on Bilibili and TikTok, as reflected not only in higher PEMAT-T and VIQI-sum scores, but also in various sub-scores (such as VIQI-2 to VIQI-4, PEMAT-U, and PEMAT-A), with only VIQI-1 showing no significant difference (Table 3). In addition, according to Table 4, videos uploaded by professionals scored higher than those by Non-professionals across all three quality assessment tools, indicating a positive impact of professional background on video quality.

**Table 3:** Quality analysis of videos about CPR on YouTube/Bilibili/TikTok.

Platform scores	Total (*N* = 239)	YouTube (N1 = 80)	Bilibili (N2 = 72)	TikTok (N3 = 87)	*P*
PEMAT-T	67.0 (34.0, 100.0)	83.0 (73.5, 88.0)^†‡^	35.0 (27.0, 39.0)^‡^	71.0 (60.0, 82.0)	<0.001
PEMAT-U	73.0 (41.0, 83.0)	84.5 (78.0, 100.0)^†‡^	35.0 (28.0, 40.8)^‡^	73.0 (64.0, 82.0)	<0.001
PEMAT-A	67.0 (39.0, 83.0)	87.5 (67.0, 100.0)^†‡^	34.0 (34.0, 50.0)^‡^	67.0 (33.0, 100.0)	<0.001
VIQI-sum	13.0 (11.0, 15.0)	15.0 (13.0, 17.0)^†‡^	11.0 (10.0, 14.0)^‡^	13.0 (11.0, 15.0)	<0.001
VIQI-1	3.0 (2.0, 4.0)	2.0 (1.0, 3.0)^‡^	2.0 (1, 2.0)^‡^	4.0 (3.0, 5.0)	<0.001
VIQI-2	4.0 (3.0, 5.0)	5.0 (4.0, 5.0)^†‡^	4.0 (3.0, 5.0)	4.0 (3.0, 5.0)	<0.001
VIQI-3	2.0 (1.0, 3.0)	3.0 (3.0, 4.0)^†‡^	1.0 (1.0, 2.0)	2.0 (1.0, 3.0)	<0.001
VIQI-4	4.0 (4.0, 5.0)	5.0 (4.0, 5.0)^†‡^	4.0 (4.0, 5.0)	4.0 (3.0, 5.0)	<0.001
GQS	4.0 (3.0, 5.0)	4.0 (3.0, 5.0)	4.0 (3.0, 4.0)	3.0 (3.0, 4.0)	0.068

^†^*P* < 0.05 vs. Bilibili; ^‡^*P* < 0.05 vs. TikTok. CPR, Cardiopulmonary Resuscitation; PEMAT, Patient Education Material Assessment Tool; VIQI, Video Information and Quality Index; GQS, Global Quality Score.

**Table 4:** Quality analysis between professionals and non-professionals.

Platform scores	Professionals (*N* = 82)	Non-professionals (*N* = 157)	*P*
PEMAT-T	82.0 (65.5, 88.0)	59.0 (36.0, 77.0)	<0.001
PEMAT-U	82.0 (67.0, 100.0)	64.0 (37.0, 79.0)	<0.001
PEMAT-A	100.0 (52.5, 100.0)	50.0 (33.0, 67.0)	<0.001
VIQI-sum	15.0 (13.0, 17.0)	12.0 (10.0, 14.0)	<0.001
VIQI-1	3.0 (2.0, 4.0)	3.0 (2.0, 4.0)	0.658
VIQI-2	5.0 (4.0, 5.0)	4.0 (3.0, 5.0)	<0.001
VIQI-3	3.0 (2.0, 3.0)	2.0 (1.0, 3.0)	<0.001
VIQI-4	5.0 (4.0, 5.0)	4.0 (3.0, 5.0)	<0.001
GQS	4.0 (3.0, 5.0)	3.0 (3.0, 4.0)	<0.001

Professionals: Professional Individuals and Professional Institutions.

Non-professionals: Non-professional Individuals and Non-professional Institutions. PEMAT, Patient Education Material Assessment Tool; VIQI, Video Information and Quality Index; GQS, Global Quality Score.

### Correlation analysis

**Figure 4:**
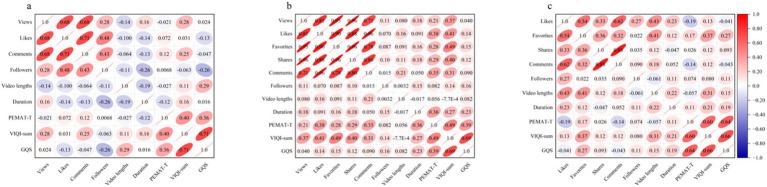
Pearson correlation analysis between scoring tool and viewer interaction on YouTube/ Bilibili/ TikTok. **(a)** Correlation between scoring tool and audience interaction on YouTube. **(b)** Correlation between scoring tool and audience interaction on Bilibili. **(c)** Correlation between scoring tool and audience interaction on TikTok. Significant level: 0.05. |*r*| ≤ 0.2 no relationship; 0.2 < |*r*| ≤ 0.4 weak relationship; 0.4 < |*r*| ≤ 0.6 moderate relationship; 0.6 < |*r*| ≤ 0.8 strong relationship; |*r*| > 0.8 very strong relationship. ^*^Excluded 5 YouTube videos with disabled comments. PEMAT, Patient Education Material Assessment Tool; VIQI, Video Information and Quality Index; GQS, Global Quality Score.

No significant strong positive correlation was found overall between video quality scores and viewer interaction metrics (Figure 4). Among all platforms, only on Bilibili was a moderate correlation observed between the VIQI-sum score and the number of likes (*r* = 0.41) and favorites (*r* = 0.49) (Figure 4b). In the overall analysis across the three video platforms and three scoring tools, comments were the only viewer interaction metric that consistently showed a stable positive correlation with all scoring tools. In contrast, followers, view lengths and duration showed almost no correlation with video quality scores. In addition, there was also a positive correlation among the three quality scoring tools, with a strong correlation observed between VIQI-sum and GQS, suggesting consistency between the two in reflecting overall video quality.

## Discussion

With the rapid development of social media platforms, short videos have become an important vehicle for health education, enabling the public to access medical information more conveniently (25). However, studies across different fields have shown that the overall quality of health-related videos is highly variable, and there is a broad academic consensus on the need to improve their scientific accuracy and standardization. In cardiovascular health education—covering topics from CPR to secondary prevention and rehabilitation—content quality issues remain common (17). Among them, CPR, as a cornerstone of emergency care and a vital life-saving skill for the general public, warrants particular attention. However, systematic quality assessment of short videos in critical care—especially those focusing on CPR—are scarce, and comparative analyses across the three major platforms, YouTube, Bilibili, and TikTok, are lacking. This gap is important, as low-quality or inaccurate CPR content could mislead viewers, delay timely intervention, and even jeopardize lives. This study systematically evaluates CPR-related videos across these platforms, aiming to provide actionable evidence to improve the scientific rigor and reliability of emergency education videos on social media, with potential benefits for public health.

As the influence of short videos continues to expand in the field of health communication, how to balance the professionalism and accessibility of the content to promote effective learning for the public has become an important topic for future health communication research. This study is the first comprehensive evaluation of CPR videos on YouTube, Bilibili, and TikTok. With the development of the Bilibili and TikTok platforms, the number of related videos has increased year by year, especially TikTok has grown significantly. Our detailed statistical analysis shows that YouTube and Bilibili videos lengths are significantly longer than TikTok (57.0 (21.0, 416.0) seconds for TikTok), which correlates with platform-specific time limits and previous literature (26). Longer videos tend to better convey complex medical information, whereas shorter videos may more effectively capture attention and facilitate rapid dissemination (27, 28). On platforms like TikTok, characterized by fast content turnover, concise videos can quickly convey key messages, reduce cognitive load, and enhance viewer engagement. This may explain TikTok’s higher VIQI-1 scores and larger follower counts, consistent with the findings of Liu et al. (29). There were significant differences in the identity of uploaders across platforms: TikTok had the highest proportion of professionals, especially more medical professionals in acute and critical care. Videos uploaded by professional individuals and institutions are generally of higher quality and more recognized by viewer, which may also explain one of the reasons for the high interaction of TikTok viewer. Overall, both certified uploader and platform characteristics influence video content and viewer interaction. Therefore, we suggest that all professionals actively seek platform certification, and the platform providers implement a more flexible and user-friendly verification process. Such changes would help eliminate barriers—such as restrictions based on professional titles or institutional affiliations—that currently hinder qualified experts from disseminating high-quality content.

Statistical analysis shows that YouTube displays a large number of professional videos on CPR knowledge and the use of AEDs, while TikTok mainly covers news report content. YouTube videos focus more on the principles and critical steps of CPR, which may enhance public understanding of first aid procedures, improve implementation accuracy, and ultimately reduce patient mortality. In contrast, Bilibili and TikTok—both Chinese short-video platforms—feature a substantial amount of “CPR Skills Challenge” footage, primarily recorded during competitions. While these videos demonstrate professional techniques, they often lack explanations of basic concepts and supplementary information, which likely contributes to their relatively low audience engagement. Although these videos may be engaging, they contribute little to enhancing the public’s fundamental first aid skills. Furthermore, a notable gap exists in pediatric and infant CPR content across all three platforms, particularly on TikTok. Given the critical importance and real-world applicability of pediatric and infant CPR, professional organizations should increase the production and dissemination of related educational videos to ensure that parents are better prepared in emergencies and to help prevent avoidable tragedies. To further address the scarcity of pediatric CPR content, we recommend three actionable steps. First, collaborate with organizations such as regional pediatric associations to develop high-quality, social media-tailored pediatric CPR content. Second, partner pediatric medical institutions with social media creators, leveraging their audience insights to turn professional guidelines into accessible, shareable material. Third, integrate pediatric CPR into school curricula and community programs, encouraging participants to share their learning online to amplify reach. These steps would strengthen pediatric CPR education across platforms.

We employed three validated assessment tools—PEMAT, VIQI, and GQS—to comprehensively evaluate video quality. The findings indicate that demonstrated significantly higher overall quality and reliability compared with those on Bilibili and TikTok. In contrast, TikTok videos are limited by their short video lengths and low information content, while Bilibili have a higher proportion of non-professional individuals. Furthermore, both platforms lack rigorous video review mechanisms, which likely contributed to their overall lower quality scores. The findings indicate that non-professional uploaders and the absence of robust review mechanisms are key contributors to the low quality of videos. Platforms should strengthen the qualification of uploaders and improve the content monitoring mechanism to improve the quality of healthy videos.

This study also found a weak correlation between video quality and viewer interaction (e.g., likes and comments), consistent with previous research (30). Viewer and platform algorithms preferred high-heat, short-lengths videos over professional and educational content, resulting in high quality health videos struggling to gain appropriate attention. In the future, platform recommendation algorithm should be optimized to prioritize, evidence-based health education videos while balancing content quality with viewer experience. In addition, there is a significant positive correlation among the three scoring tools, especially the strong correlation between VIQI-sum and GQS, indicating that these measures may partially reflect video popularity (31). The combined use of multiple assessment tools is recommended in future research to enhance the accuracy and objectivity of quality evaluations. To improve the quality of CPR short videos, platforms should implement stricter content quality controls and strengthen both the support for and certification of qualified professionals. Platforms should enhance the auditing and supervision of health education videos to limit the dissemination of misleading information. In parallel, governmental and relevant regulatory bodies should conduct regular monitoring and removal of low-quality or misleading content to safeguard the credibility and accuracy of public health information.

Several limitations of this study should be acknowledged. First, although anonymous device access was employed and no user accounts were logged in, the influence of personalized recommendation algorithms could not be entirely eliminated. Second, the exclusion of videos without subtitles may have resulted in the omission of some high-quality content. Third, although three evaluators were used to independently score and standardize the disagreement, the scoring tool itself is still subjective and cannot avoid potential systematic bias. Future research could develop a more comprehensive and objective content quality assessment tool, combining with AI technology to improve assessment efficiency and accuracy. Finally, the sample size was limited, and some videos were uploaded earlier. With the evolution of technology and platforms, the quality of CPR videos may be further improved in the future, which deserves continuous attention and evaluation.

## Conclusion

CPR-related videos on social media platforms have contributed to the popularization of first aid knowledge and skills among the public. TikTok’s short-form, highly interactive videos attract substantial viewer attention, while YouTube and Bilibili offer longer, more professional content with higher quality ratings. Nonetheless, video quality across all platforms remains suboptimal, particularly regarding content completeness and professionalism. Therefore, there is an urgent need to encourage more professionals to participate in the production of CPR videos, while platforms should strengthen their content review and recommendation mechanisms to ensure that high-quality videos are fully exposed. By optimizing the algorithms, the platforms are able to push more scientific and accurate first aid knowledge to users, further enhancing the public’s first aid awareness and practical operation ability. In conclusion, improving the overall quality of CPR-related content and minimizing public exposure to misleading information is essential for advancing social first-aid capacity and safeguarding public health—an objective that requires coordinated efforts from multiple stakeholders.

## Data Availability

The datasets presented in this article are not readily available because the datasets generated and analyzed during the current study are not publicly available, but may be made available by the corresponding author upon reasonable request and subject to individual consideration. Requests to access the datasets should be directed to Chuanliang Pan, cl.pan@foxmail.com.
