# Supplementary material for: Professional content analysis and quality assessment of cardiopulmonary resuscitation educational videos on social media platforms: a comparative study of YouTube, BiliBili, and TikTok
Source: Front Public Health. 2025 Sep 15;13:1657233. doi: 10.3389/fpubh.2025.1657233 (PMC12477029; doi:10.3389/fpubh.2025.1657233)
Supplement: Supplementary file 1 [file Supplementary_file_1.docx]

| Multimedia Appendix 1. Classification of video uploaders and content. | |
| --- | --- |
| **Video Source** | |
| Professional Individuals | Individuals with real name recognition and professional accreditation in the medical field, including doctors, nurses, paramedics, and other healthcare professionals. |
| Non-professional Individuals | Individuals without real name recognition or professional accreditation in the medical field. |
| Professional Institutions | Institutions with professional accreditation in the medical field, including hospitals, medical schools, emergency centers, and other healthcare institutions. |
| Non-professional Institutions | Institutions without professional accreditation in the medical field, including newspapers, TV stations, online media, and other non-professional groups. |
| **Source of Professional Individuals** | |
| Doctors Specializing in Emergency Medicine | Including emergency department doctors, intensive care doctors, and other doctors working in emergency medicine-related fields. |
| Doctors in Other Fields of Modern Medicine | Including doctors in other fields such as surgery, internal medicine, pediatrics, anesthesiology, etc. |
| First aiders and care personnel | Including first aiders, nurses, care staff, and other professionals with first aid skills. |
| Other Healthcare Professionals | Including epidemiologists, technicians, basic research experts, and other healthcare professionals. |
| **Video Content** | |
| CPR Knowledge | Including the basic principles steps, detailed demonstration of CPR, indications, and contraindications of CPR. |
| Case Analysis and Practice | Providing real or simulated CPR cases, analyzing key points and precautions during operations. |
| Application of Automated External Defibrillators | Introduction to equipment that may be used during CPR, such as the use of automated external defibrillators (AEDs). |
| CPR Skills Challenge Footage | Footage showcasing individuals or groups performing CPR techniques in a timed or competitive setting. |
| News and Reports | News reports or stories about successful CPR cases. |
