# Supplementary material for: Professional content analysis and quality assessment of cardiopulmonary resuscitation educational videos on social media platforms: a comparative study of YouTube, BiliBili, and TikTok
Source: Front Public Health. 2025 Sep 15;13:1657233. doi: 10.3389/fpubh.2025.1657233 (PMC12477029; doi:10.3389/fpubh.2025.1657233)
Supplement: Supplementary file 2 [file Supplementary_file_2.docx]

Multimedia Appendix 2. The specific scoring methods and characteristics of each assessment tool.

**Table S1.** Video Information and Quality Index (VIQI)

| **VIQI score** | **VIQI-1** | Flow of information |  |
| --- | --- | --- | --- |
|  | **VIQI-2** | Information accuracy |  |
|  | **VIQI-3** | Quality (videos including one point for each one image, animation, interview, video captions and summary) |  |
|  | **VIQI-4** | Precision (level of coherence between video title and content) |  |
|  | **Total VIQI Score** | Sum of all VIQI scores |  |
|  | *The VIQI scale contains four evaluation criteria, and the videos were scored using 5-point Likert scale for each criterion. | | |

**Table S2.** Global Quality Score (GQS) Criteria

| **GQS score** | **Score** | **Description** |
| --- | --- | --- |
|  | **1** | Poor quality, poor flow, most information missing, not useful for education |
|  | **2** | Generally poor quality and flow, of limited use to patients because only some information is present but many important topics missing |
|  | **3** | Moderate quality, suboptimal flow, somewhat useful for patients as some important information is adequately discussed but others poorly discussed |
|  | **4** | Good quality, generally good flow, useful to patients because most relevant information is covered but some topics not covered |
|  | **5** | Excellent quality and flow, highly useful to patients |
|  | *The GQS evaluation was performed which scored the videos in a range between 1 (poor quality) to 5 (excellent quality). | |

**Table S3.** Patient Education Materials Assessment Tool (PEMAT)

The detailed information of the Patient Education Materials Assessment Tool (PEMAT) can be found at the website <https://www.ahrq.gov/health-literacy/patient-education/pemat.html>. If you have Excel, you can also use the PEMAT Auto-Scoring Form, a form that will automatically calculate PEMAT scores once you enter your ratings. Download the **PEMAT Auto-Scoring Form** ([Excel](https://www.ahrq.gov/sites/default/files/wysiwyg/professionals/prevention-chronic-care/improve/self-mgmt/pemat/pemat_form.xls), 235 KB).
